# Secretory IgA and course of COVID-19 in patients receiving a bacteria-based immunostimulant agent in addition to background therapy

**DOI:** 10.1038/s41598-024-61341-7

**Published:** 2024-05-15

**Authors:** Mikhail Kostinov, Oksana Svitich, Alexander Chuchalin, Valery Osiptsov, Ekaterina Khromova, Natalya Abramova, Vitaly Tatevosov, Anna Vlasenko, Vilia Gainitdinova, Dmitrij Pakhomov, Kirill Mashilov, Tatyana Ospelnikova, Natalya Mihajlova, Valentina Polishchuk, Ekaterina Kurbatova, Aristitsa Kostinova

**Affiliations:** 1 Department of Epidemiology and Modern Vaccination Technologies, Institute of Professional Education, I.M. Sechenov First Moscow State Medical University, Moscow, Russian Federation; 2 Laboratory of Preventive Vaccination and Immunotherapy of Allergic Diseases, I.I. Mechnikov Research Institute of Vaccines and Sera, Moscow, Russian Federation; 3 I.I. Mechnikov Research Institute of Vaccines and Sera, Moscow, Russian Federation; 4 Department of Hospital Therapy of the Faculty of Pediatrics, Pirogov Russian National Research Medical University (Pirogov Medical University, Moscow, Russian Federation; 5 The Main Military Clinical Hospital of the National Guard Troops of the Russian Federation, Moscow, Russian Federation; 6 Laboratory of Molecular Immunology, I.I. Mechnikov Research Institute of Vaccines and Sera, Moscow, Russian Federation; 7 Department of Medical Cybernetics and Informatics Novokuznetsk State Institute for Advanced Medical Education of Physicians, Branch Campus of the Russian Medical Academy of Continuous Professional Education, Novokuznetsk, Russian Federation; 8 Pulmonology Department, N.V. Sklifosovsky Institute of Clinical Medicine, I.M. Sechenov First Moscow State Medical University, Moscow, Russian Federation; 9 National Research Centre for Epidemiology and Microbiology Named After the Honorary Academician N.F. Gamaleya, Moscow, Russian Federation; 10 Laboratory of Therapeutic Vaccines, I.I. Mechnikov Research Institute of Vaccines and Sera, Moscow, Russian Federation

**Keywords:** COVID-19 hospitalization, Mucosal immunity, sIgA, Bacterial ligands during COVID-19, Microbial-based immune therapy in COVID-19, Diagnostic markers, Immunological disorders, Infectious diseases, Respiratory tract diseases, Immunotherapy, Infectious diseases, Mucosal immunology, Vaccines

## Abstract

Mucosal immunity plays a major role not only in the prevention but probably also in the outcomes of COVID-19. An enhanced production of secretory immunoglobulin A (sIgA) might contribute to the activation of the immune response mechanisms. To assess the levels of sIgA produced by epithelial cells in the nasal and pharyngeal mucosa and those measured in salivary gland secretions and to study the course of COVID-19 following the combined scheme of intranasal and subcutaneous administration of a bacteria-based immunostimulant agent. This study included 69 patients, aged between 18 and 60, who had moderate COVID-19 infection. They were divided into two groups: Group 1 (control group) included 39 patients who received only background therapy, and Group 2 was made up of 30 patients who received background therapy in combination with the Immunovac VP4 vaccine, a bacteria-based immunostimulant agent, which was given for 11 days starting from the day of admission to hospital. The levels of sIgA were measured by ELISA in epithelial, nasal and pharyngeal swabs, and salivary gland secretions at baseline and on days 14 and 30. The combined scheme of intranasal and subcutaneous administration of the Immunovac VP4 vaccine in the complex therapy of patients with COVID-19 is accompanied by increased synthesis of sIgA in nasal and pharyngeal swabs, more intense decrease in the level of C-reactive protein (CRP) and reduction in the duration of fever and length of hospitalization compared to the control group. Prescribing a immunostimulant agent containing bacterial ligands in complex therapy for COVID-19 patients helps to enhance mucosal immunity and improves the course of the disease.

## Introduction

The mucosal immunity plays a crutial role in preventing droplet infections, including SARS-CoV-2. In case of SARS-CoV-2, infection is, however, facilitated by some structural features of the virus and the fact that it engages angiotensin-converting enzyme 2 (ACE2) as the primary receptor and employs the transmembrane serine protease 2 (TMPRSS2) for protein priming^1^. The induction of mucosal immunity is in the future likely not only to become a strategy in preventive vaccination against particular infections, e.g. SARS-CoV-2 infection, but also a treatment strategy, i.e. a tool for restoration of a balanced profile of immunocompetent cells, which are responsible for limiting the spread of infection and localizing it at the site of entry at earlier stages. Despite ongoing research of the mechanisms of mucosal immunity in viral infections, in particular coronavirus infection, the approaches to immune therapy and the role of immunobiological medications in the activation of mucosal immunity during the active inflammation stage have not been fully investigated^2^.

The majority of microbial-based immunomodulatory agents demonstrate highly favorable safety profile and effectiveness in reducing of respiratory infectionsas well as the need for antibiotics and other medications while maintaining the treatment^3–7^. It is believed that the recognition of bacterial antigens included in such formulations by dendritic cells activates immune response and stimulates the production of antibodies by B-cells, which is accompanied by the enhancement of phagocytic activity of macrophages and polymorphonuclear neutrophils as well as an increased production of lysozyme and secretory immunoglobulin A (sIgA)^8,9^. Bacteria-based immunomodulatory agents induce the polarization of immune response, mainly type Th1, increase in NK cytotoxicity and an enhanced expression of TLR2, TLR4, and TLR9^10^ that is why they are especially used in the treatment of the respiratory tract diseases such as obstructive pulmonary disease and asthma^11–14^. The results of the studies investigating the efficacy of immunomodulatory agents in COVID-19 infection are, however, scarce, anecdotal, and sometimes not evidence-based. It has been supposed that products containing bacterial ligands used as part of a combination treatment help maintain high levels of sIgA throughout the treatment period^15^. Enhanced production of sIgA detected in nasal epithelial secretion may activate the mechanisms of mucosal immune response and contribute to a favorable course and outcome of COVID-19 disease; there may also be a correlation between sIgA levels and the clinical symptoms^16–18^. A previously published study on the use of bacterial ligands (Immunovac VP4) in the comprehensive treatment of patients with COVID-19 with moderate damage of lung tissue showed that in the group of patients receiving this drug, normalization of temperature was reached faster and the length of hospital stay was significantly reduced^19^. The obtained clinical effect of the administration of Immunovac VP4 is associated with the activation of sIgA synthesis in mucous membranes from various loci of the upper respiratory tract. The immunomodulatory drug in that study was prescribed intranasally (IN) and per os like most topical and systemic bacterial lysates (“IRS-19”, “Bronchovaxone”, “Ribomunil”, etc.). Since COVID-19 in patients is accompanied by profound changes in various parts of the immune system, it was of interest to evaluate the effect of combined regimen of intranasal and parenteral administration (subcutaneous injection) of Immunovac VP4. It should be noted that all these methods are eligible to be used and indicated in the instruction for the drug^20^.

### Objective

To study the level of the secretory component IgA of epithelial cells from the nasal mucosa, pharyngeal scraping and in secretions of salivary glands and the course of COVID-19 in patients with a combined regimen of intranasal (IN) and subcutaneous (SC) administration of the bacterial ligand based immunostimulant Immunovac VP4.

## Materials

### Clinical study design

The primary objectives were to evaluate the changes in sIgA levels taken from various loci of the upper respiratory tract in COVID-19 patients from the moment of admission to the hospital, on days 14 and 30 and to assess the impact of a bacteria-based immunostimulant on the secretion of sIgA, the duration of fever, the number of hospital days, and the CRP level.

A total of 69 patients were included in the study. They were divided into the following groups: Group 1 (n = 39) included patients who received only basic therapy of COVID-19, and Group 2 (n = 30) was made up of patients who received basic therapy in combination with Immunovac VP4—a bacteria-based vaccine.

This was a Phase IV controlled non-randomized postmarketing study. It was conducted in a dedicated COVID-19 hospital in Moscow (Russian Federation). Patients were included into the study after medical tests, physical examination and an assessment of the inclusion and exclusion criteria as well as in accordance with indications and contraindications for Immunovac VP4. Patients were followed up for a minimum of 30 days. All treatment information, physical examination findings and test results were reported using standard medical records (individual patient documentation).

### Legal and ethical conduct of the study

The severity of COVID-19 in patients was assessed according to the temporary guidelines “Prevention, diagnosis and treatment of new coronavirus infection (COVID-19)” of the Russian Ministry of Health dated October 26, 2020. Treatment of patients in the hospital was carried out in appliance with the above clinical guidelinesand clause 20 "Voluntary Informed Consent to Medical Intervention and Refusal of Medical Intervention" (Federal Law No. 323-ФЗ, dated November 1, 2011 "On Fundamental Healthcare Principles in the Russian Federation" (as amended on April 3, 2017).

The study protocol №8 was approved on November 26, 2020 by the local Ethics Committee of the Federal State Budgetary Scientific Institution I.I. Mechnikov Research Institute of Vaccines and Sera (Russian Federation). The study was conducted in accordance with the Declaration of Helsinki, the International Council for Harmonization's Good Clinical Practice guideline and Russian regulatory requirements. Written informed consent was obtained from patients prior to their enrollment in the study.

## Patients

A total of 69 inpatients, aged between 18 and 60, hospitalized from November 30, 2020 till May 30, 2021 with a confirmed COVID-19 infection with moderate lung involvement were included in the study. Hospitalization lasted from 14 to 21 days. SARS-CoV-2 infection was confirmed by PCR of nasopharyngeal swabs and/or clinical and X-ray findings (all patients had computed tomography [CT] signs of lung injury such as ground-glass opacities and areas of consolidation consistent with grade 2 CT scan [25%-50% lung involvement]). The COVID-19 patients included in the study met all the inclusion criteria and did not meet the exclusion criteria. They received basic therapy which was selected according to the severity of their disease and as recommended by the clinical guidelines developed by the Ministry of Health of the Russian Federation. It included Favipiravir 200 mg (standard regimen), enoxaparin 0.4 mg/day, subcutaneously, dexamethasone 8–12 mg/day, and tocilizumab 400 mg/day (for patients with CRP ≥ 60 mg/L).

The patients were randomly assigned to two groups. Group 1 (control group) consisted of 39 patients (Table 1). These patients received only basic background therapy.

**Table 1:** Clinical characteristics of hospitalized patients with COVID-19 with moderate lung damage.

Parameter	Total (n = 69)	
	1 gr. (n = 39) Background therapy	2 gr. n = 39 Background therapy + Immunovac VP4
Age, years	42 (33–54)	42 (37–45) p = 0.79
Men/women	26/13	22/8 p = 0.33
Number of days from the onset of illness	5 (4–8)	5(3–7) p = 0.63
BMI, kg/m^2^	29 (26–33)	28 (25–32) p = 0.77
Respiration rate per minute	23 (22–25)	24 (23–26) p = 0.81
Heart rate per minute	87 (83–101)	88 (84–103) p = 0.85
SpO_2_, %	92 (91–94)	92 (90–94) p = 0.32
CRP, mg/l	69 (39–89)	73 (41–96) p = 0.64
Fibrinogen, g/l	5.5 (4.6–5.9)	5.4 (4.4–5.7) p = 0.42
D-dimer	0.6 (0.5–0.8)	0.6 (0.4–0.9) p = 0.67
CT, % of the lung damage	43 (38–49)	44 (39–51) p = 0.47

*BMI* body mass index, *SpO2* blood oxygen saturation, *CRP* C-reactive protein, *CT* computed tomography of the chest organs.

Group 2 was comprised of 30 patients. These patients received Immunovac VP4 vaccine, a bacteria-based immunostimulant, as an add-on to the background therapy. This vaccine was given starting on day 1 of hospitalization after careful consideration of all indications and contraindications as per the package insert.

These groups of patients were matched by body mass index, amount of impaired lung parenchyma, and laboratory findings.

Samples were also taken from different compartments of the upper respiratory tract of healthy COVID-19 unvaccinated healthcare workers who had not been exposed to SARS-CoV-2 (n = 10). The study parameters were measured in these samples; median values were calculated and considered as median reference values.

### Inclusion criteria

Inpatients aged between 18 and 60 with confirmed COVID-19 infection, i.e. SARS-CoV-2 detected in a nasopharyngeal swab by PCR and/or clinical and X-ray confirmation (all patients had CT signs of lung injury such as ground-glass opacities and areas of consolidation consistent with grade 2 CT scan [25–50% lung involvement]), and signed and dated informed consent.

### Exclusion criteria

Patients were excluded if they met any of the following criteria: lung abscess, pleural empyema, active tuberculosis; severe birth defects or serious chronic disorders, including exacerbations/decompensation of chronic disorders, such as pulmonary, liver, renal, cardiovascular, neurological, or mental disorders, malignancies within the last five years, metabolic diseases; HIV or hepatitis B or C; use of immunoglobulin or blood transfusion within the last three months prior to the start of the study; long use (more than 14 days) of immunosuppressive or other immunomodulatory drugs within six months prior to the start of the study; any known or suspected immunosuppressive or immunodeficiency disorder or active autoimmune disease; any vaccination within the last month; pregnancy or lactation; simultaneous participation in another clinical study; or the patient’s inability to comply with the study protocol requirements (as judged by the investigator).

## Studied drug

### Immunovac VP4 vaccine

This is a polyvalent vaccine based on the antigens of opportunistic microorganisms (mixture of water-soluble antigens extracted from *Staphylococcus aureus, Klebsiella pneumoniae, Proteus vulgaris,* and *Escherichia coli*). This product is approved for subcutaneous use (Registration Certificate # ЛCP-001294/10 issued by the Ministry of Health of the Russian Federation on February 24, 2010) as well as nasal and oral use (Registration Certificate # ЛCP-001293/10 issued by the Ministry of Health of the Russian Federation on February 24, 2010). It is manufactured by Scientific and Production Association for Immunological Preparations “Microgen”, a federal state unitary enterprise (Ufa, Russian Federation).

### Pharmacological properties

It is a bacteria-based immunostimulant. Its mechanism of action is due to the activation of the key effectors of innate and adaptive immunity. This vaccine enhances phagocytic activity of macrophages, optimizes T-cell counts and functional activity of lymphocyte subsets (CD3 + , CEM, CD8 + , CD16 + , and CD72 +), programs CD4 + T-cells to proliferate and differentiate into Th1 cells, stimulates the production of IFN-γ and IFN-α, and improves the production of immunoglobulin isotypes by inhibiting IgE synthesis and inducing IgG, IgA, and sIgA synthesis. It induces the production of antibodies to four opportunistic microorganisms whose antigens are included in the composition. It also provides cross protection against *Streptococcus pneumoniae, Haemophilus influenzae* and other pathogens due to the existence of common antigen components. In terms of clinical outcomes, vaccination reduces the rate of acute infections, duration of infection, severity of symptoms, risk of exacerbation of chronic diseases, and the amount of medication treatment.

The vaccine according to the instructions for its use can be administered depending on one of suggested combined regimens: intranasally and orally (per os), as well as intranasally and subcutaneously Immediately prior to use, 2 mL of solvent (0.9% sodium chloride for injection or boiled water brought to 18–25 °C) is added to the vial with a syringe, and the contents is mixed. The product is instilled into the nasal cavity using a medical dropper. For oral use, the required amount of vaccine is drawn from a vial with a syringe and then transferred into a spoon.

### Drug interactions

The product can be used with other medications as part of combination treatment. It can be administered in combination with antibiotics, antiviral, antifungal and antihistamine agents, bronchodilators, corticosteroids, and β-adrenoceptor agonists. Patients who receive immune therapy or immunoprophylaxis with Immunovac VP4 should not receive any other immunomodulatory agents within one month prior to this course of therapeutic or preventive treatment and within three months after its completion.

### Schedule, dose and timing for vaccination in the study

When prepared, the solution of Immunovac VP4 was administered to patients at a dose of 2 drops (1 mg) in each nostril daily and subcutaneously every other day at doses 0.05 (0.5), 0.1 (1.0), 0.2 (2.0), 0.2 (2.0), 0.3 (3.0), and 0.3 (3.0) from day 1 to day 11 of hospital stay according to the instruction for use for the combined intranasal and subcutaneous regimen.

## Methods

For all patients, demographic data, body mass index, symptoms of the disease, physical examination findings, results of laboratory tests (complete blood count, C-reactive protein, and blood coagulation profile) and other investigations (chest computed tomography), and concomitant diseases were assessed.

The severity of respiratory failure was defined by the blood oxygen saturation level measured by pulse oximetry (SpO_2_). Patients' nutritional status was assessed by body mass index, which was calculated using the standard formula: Body mass index = weight (kg)/height (m^2^). Pulse oximetry was performed using a pulse oximeter (series MD300C).

Lung CT was performed on a spiral CT scanner Aquilion TSX-101A (Toshiba Medical Systems, slice thickness 1 mm, pitch 1.5) on admission and after 10 days of treatment.

### Sampling

In study groups 1 and 2, samples were taken from different compartments of the upper respiratory tract: nasal mucosal epithelial scrapings, pharyngeal epithelial scrapings, and salivary gland secretions. Saliva was collected early in the morning before patients brushed their teeth and had a meal. Saliva was collected passively without any forceful coughing under supervision of a physician^21–23^. Sampling was performed in two steps: on study day 1 before study treatment was administered, on study day 14, and subsequently 30 days after the start of treatment.

Clinical laboratory tests, including CRP, were done in accordance with the institutional standards and patients’ condition.

Cytobrush sampling was performed in all patients to determine protein levels. Samples were collected using a type D brush (Yunona, Russian Federation) into three Eppendorf Tubes with sodium chloride solution. The tubes were centrifuged at 2000*g* for about 5 min to sediment the epithelial cells and then refrigerated at + 2–4 °C until shipment to the laboratory, where the samples were examined within 24 h of collection.

Levels of sIgA in all biological fluids were measured by enzyme-linked immunosorbent assay (Vector Best, Russian Federation). Plates were read using a Multiskan Ascent ELISA microplate photometer (Thermo Electron Corporation, Finland). Levels of immunoglobulins were measured by enzyme-linked immunosorbent assay based on a two-step sandwich enzyme immunoassay using monoclonal antibodies (mAb) against the secretory component linked to alpha chain of IgA. Standards with known concentrations of sIgA and the samples were added to the wells of a plate coated with an anti-sIgA mAb. The plate was then incubated according to the test kit instructions. The intensity of developing color is proportional to the concentration of sIgA in the sample. The concentration of sIgA was calculated using the standard curve and the measured optical density values.

These tests were performed using certified equipment provided by the Research Equipment Sharing Center of the Federal State Budgetary Scientific Institution I.I. Mechnikov Research Institute of Vaccines and Sera.

### Statistics

The normality of distribution of the quantitative variables was tested using the Shapiro Wilk's normality test. Most variables were found to have a non-normal distribution, therefore, descriptive statistics for quantitative variables included median and interquartile range, Me (Q1–Q3). The 95% confidence intervals were calculated for the differences between the medians at the two time points.

Changes over time in sIgA levels were compared between the study groups by using a linear mixed-effects model, where group and time point were fixed factors, and patients were random factors. This model was created in the lme4 package^24^. When the model was created, goodness-of-fit tests (normality of distribution and homogeneity of variance in residuals) were conducted using the DHARMa package^25^. If these goodness-of-fit tests showed some problems, a Box-Cox transformation was applied to the initial dataset, then a corrected model was built and goodness-of-fit tests were run on the transformed data. These are modelling results for the pooled data obtained at three time points by applying type III ANOVA with Kenward-Roger approximation for degrees of freedom, these tests were performed using the lmerTest package^26^. All post-hoc tests were performed using corresponding contrasts in the calculated linear mixed-effects model with a Benjamini–Krieger–Yekutieli correction^27^.

Individual quantitative variables were compared between the study groups using the Mann–Whitney test. The one-sample Wilcoxon test was used to compare the medians of quantitative parameters to the expected medians.

The level of statistically significant differences was defined as p ≤ 0.05. Calculations and graphics were carried out using GraphPad Prism (v.9.3.0, license GPS-1963924) and the statistical programming environment R (v.3.6, license GNU GPL2).

## Results

Table 2 provides an analysis of changes in sIgA in the study COVID-19 patients over the period between admission to hospital and discharge and 30 days after the start of the study.

**Table 2:** Analysis of changes over time in sIgA levels in the study groups at the study time points.

Study group	sIgA at study time points, µg/L—Me(Q1-Q3)			p values for changes over time^5^
	Baseline	After 14 days	After 30 days	
Nasal swap (reference value 29.9 µg/L)				
Control^1^	91.3 (50.9–156.0)	59.0 (21.9–146.5)	30.2 (7.6–61.7)	p^0–14^ = 0.13, **p**^**0–30**^** = 0.02, p**^**14–30**^** = 0.002**
VP4^2^	77.5 (37.0–91.5)	46.7 (19.8–109.2)	107.0 (44.9–164.5)	p^0–14^ = 0.35, **p**^**0–30**^** = 0.01, p**^**14–30**^** = 0.02**
p values for groups	p = 0.08	p = 0.27	**p = 0.002**	-
LMEM^3^—Group: F = 0.7, p(63.8) = 0.42 Time: F = 0.6, p(100.4) = 0.56 **Group × Time: F = 10.8, p(100.4) = 0.001**				
Pharyngeal swab (reference value 6.5 µg/L)				
Control	6.6 (1.0–30.4)	9.4 (1.1–25.3)	7.9 (1.1–13.9)	p^0–14^ = 0.69, p^0–30^ = 0.29, p^14–30^ = 0.32,
VP4	1.0 (0.4–11.7)	9.1 (0.9–17.9)	19.6 (3.7–54.1)	p^0–14^ = 0.06, p^0–30^ = 0.09, **p**^**14–30**^** = 0.001**
p values for groups	p = 0.11	p = 0.69	**p = 0.05**	-
LMEM^4^—Group: F = 0.3, p(63.6) = 0.62 Time: F = 3.3, p(95.0) = 0.04 **Group × Time: F = 6.2, p(95.0) = 0.003**				
Salivary gland secretions (reference value 71.7 µg/L)				
Control	156.8 (64.3–234.9)	120.8 (54.3–172.9)	150.9 (119.5–192.3)	-
VP4	177.3 (94.5–234.2)	154.4 (87.8–184.1)	148.4 (93.5–202.9)	
LMEM^5^—Group: F = 0.2, p(59.8) = 0.66 Time: F = 0.8, p(95.2) = 0.46 **Group × Time:** F = 0.0, p(95.2) = 0.97				

Significant values are given in bold.

^1^Group of background therapy.

^2^Group of background therapy + Immunovac VP4.

^3^A linear mixed-effects model (LMEM) was used, where group and time point were fixed factors, and patients were random factors. These are pooled results for three time points obtained by applying type III ANOVA with Kenward-Roger approximation for degrees of freedom.

^4^Calculations were done using pre-transformed data. Data transformation was performed using the Box-Cox method (λ = − 0.12).

^5^Post-hoc tests (p values for changes over time were the values for comparison between time points, and p values for groups were the values for comparison between the two study groups at each study point) were performed using corresponding contrasts in the calculated linear mixed-effects model with a Benjamini–Krieger–Yekutieli correction.

### Levels of sIgA in salivary gland secretions

**Figure 1:**
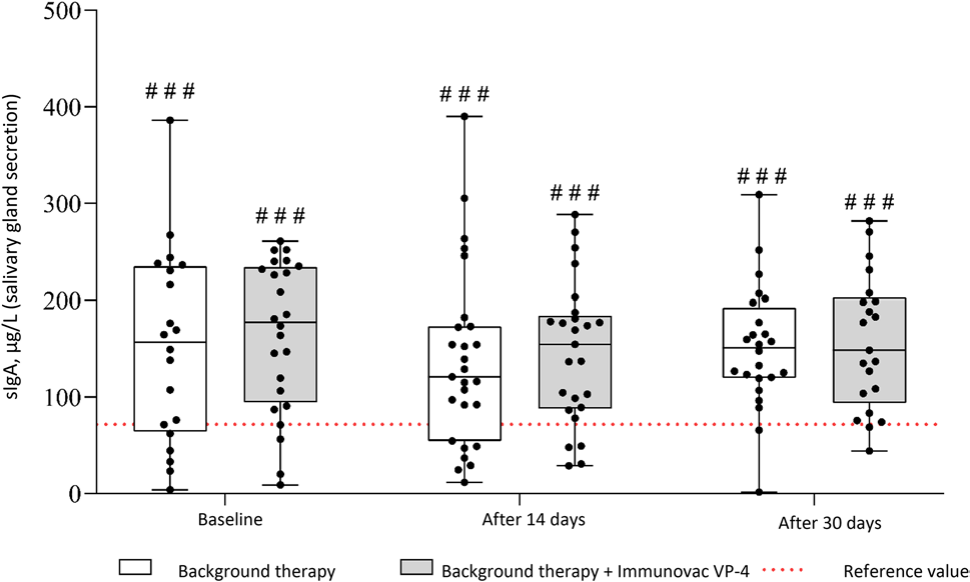
Changes over time in salivary sIgA levels in COVID-19 patients who received background therapy alone or in combination with Immunovac VP4 (at baseline and on days 14 and 30). ^###^p < 0.001 for comparison against the reference value (in healthy unvaccinated study subjects), the one-sample Wilcoxon test was used.

The study groups did not show statistically significant difference in terms of either absolute salivary sIgA levels throughout the study period or their changes from baseline (Fig. 1). Of note, in COVID-19 patients salivary sIgA levels were significantly higher than in healthy who had not had COVID-19 and unvaccinated healthcare workers over the entire study period (p < 0.001 for comparisons between the values measured at each time point in each study group and the median reference value [71.7 µg/L]).

### Levels of sIgA in pharyngeal swabs

**Figure 2:**
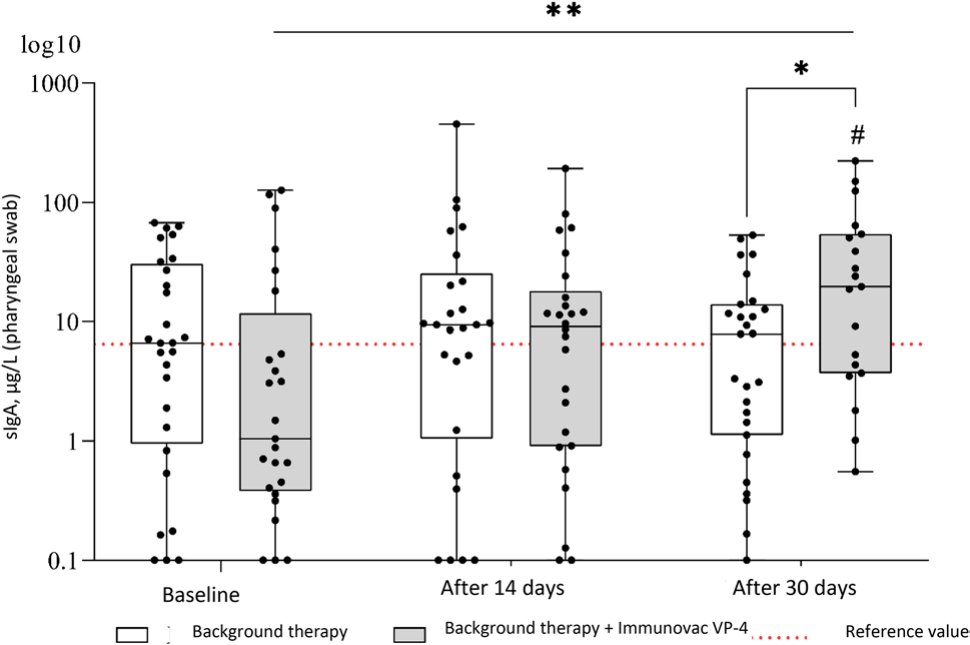
Changes over time in pharyngeal sIgA levels in COVID-19 patients who received background therapy alone or in combination with Immunovac VP4 (at baseline and on days 14 and 30). *p ≤ 0.05, **p < 0.01, a linear mixed-effects model was used, and a Benjamini–Krieger–Yekutieli correction was applied for multiple comparisons. ^#^p ≤ 0.05 for comparison against the reference value (in healthy unvaccinated study subjects), the one-sample Wilcoxon test was used.

Evaluation of pharyngeal swabs revealed significant diverse changes in the sIgA levels for patients receiving and not receiving Immunovac VP4 (F = 6.2, p(95.0) = 0.003; Fig. 2). In the control group (without use of vaccine), these levels did not show statistically significant difference from the baseline values throughout the study, whereas the patients who received Immunovac VP4 in addition to background therapy showed a significant increase from baseline in pharyngeal sIgA levels on day 30 after the start of the study (from 1.0 [0.4–11.7) µg/L to 19.6 (3.7–54.1) µg/L, p = 0.001). Thirty days after the start of the study, pharyngeal sIgA increased by 18.1 (ranging from + 0.8 to + 27.1) µg/L in patients who received Immunovac VP4 in addition to background therapy compared to 1.3 [ranging from − 6.1 to + 3.3] µg/L in patients who received only background therapy; this difference was statistically significant (p = 0.001). At baseline, the study groups did not show statistically significant difference in the pharyngeal sIgA levels (p = 0.11). Of note, the baseline levels of pharyngeal sIgA in either study group did not significantly differ from those observed in the healthy COVID-19 unvaccinated participants who had not been also infected with it (p = 0.25 and p = 0.47 for comparisons of the values in the control group and the Immunovac VP4 group, respectively, to the median reference value [6.5 µg/L]). Nevertheless, 30 days after the start of treatment, patients in the Immunovac VP4 group had higher levels of pharyngeal sIgA than the patients who received only background therapy (19.6 [3.7–54.1] µg/L *vs.* 7.9 (1.1–13.9) µg/L, p = 0.05) and healthy study subjects (p = 0.01).

### Levels of sIgA in nasal swabs

**Figure 3:**
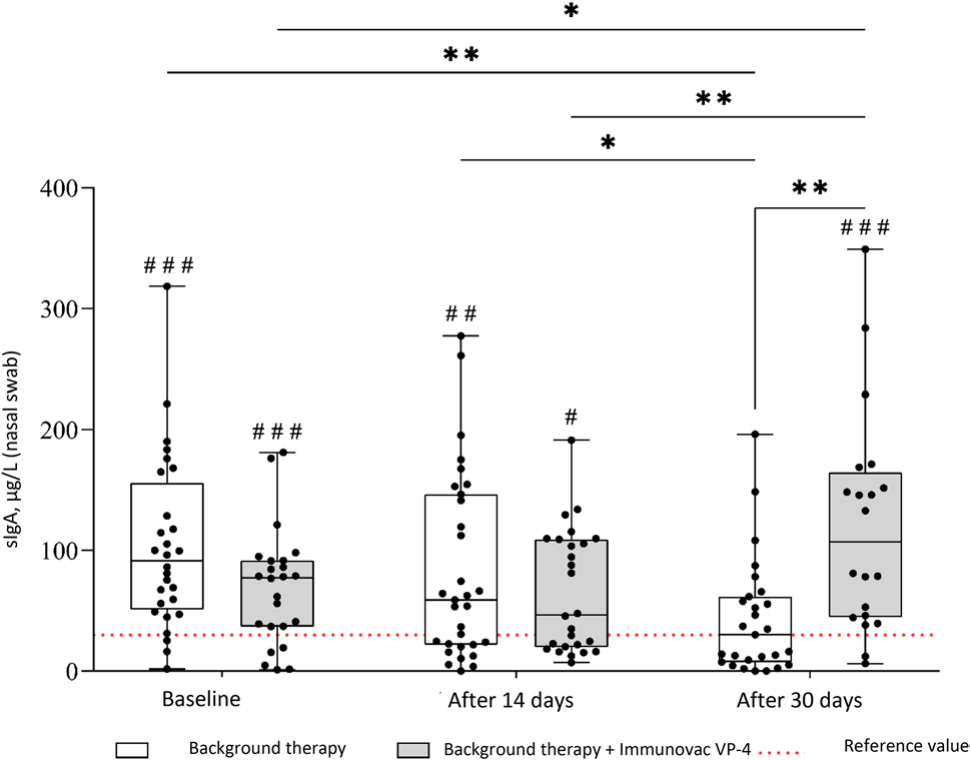
Changes over time in nasal sIgA levels in COVID-19 patients who received background therapy alone or in combination with Immunovac VP4 (at baseline and on days 14 and 30). *p ≤ 0.05, **p < 0.01, a linear mixed-effects model was used, and a Benjamini–Krieger–Yekutieli correction was applied for multiple comparisons. ^#^p ≤ 0.05, ^##^p < 0.01, ^###^p < 0.001 for comparison against the reference value (in healthy unvaccinated study subjects), the one-sample Wilcoxon test was used.

The most significant divergent changes in sIgA levels depending on the treatment administered were observed in nasal swabs (F = 10.8, p[100,4]  < 0.001, Fig. 3). At baseline, COVID-19 patients who received background therapy alone or in combination with Immunovac VP4 had similar sIgA levels in nasal swabs (p = 0.08); however, 30 days after the start of the study patients who were given Immunovac VP4 in addition to background therapy had a statistically significant increase from baseline in this parameter (from 77.5 [37.0–91.5] µg/L to 107.0 [44.9–164.5] µg/L, p = 0.02). In contrast, the control group showed a significant decrease in sIgA compared to the baseline values (from 91.3 [50.9–156.0] µg/L to 30.2 [7.6–61.7] µg/L, p = 0.002). Thus, on day 30 after the start of treatment, patients receiving Immunovac VP4 in combination with background therapy had statistically significantly higher levels of sIgA than patients who received only background therapy (p = 0.002). On day 30 of the study, the change from baseline (the difference in medians) in sIgA levels was -61.0 (ranging from − 84.3 to − 28.6) µg/L in the control group and + 29.5 (ranging from − 3.2 to + 82.7) µg/L in the Immunovac VP4 group, with this difference being statistically significant (p = 0.005). It should also be noted that at baseline nasal sIgA levels in both COVID-19 patients were higher than healthy COVID-19 unvaccinated participants who had not been also infected with it still (p < 0.001 for comparisons of the values in both study to the median reference value [29.9 µg/L]), 30 days after the start of treatment in the control group this parameter was similar to that in healthy subjects (p = 0.40) while in the Immunovac VP4 group it remained higher than in healthy subjects (p < 0.001).

**Figure 4:**
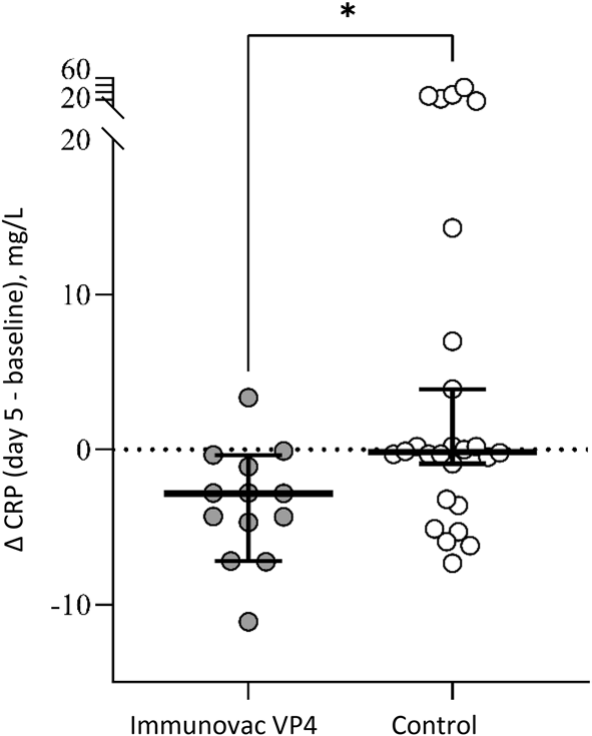
Absolute change from baseline (delta) in CRP on day 5 in the two study groups of COVID-19 patients; individual values, medians and 95% confidence intervals.

At baseline COVID-19 patients had high CRP levels, which were comparable in the study groups (p = 0.15). On day 5, CRP levels showed a statistically insignificant decrease from baseline in the control group (from 2.0 [0.3–5.5] mg/L to 1.6 [0.1–6.5] mg/L (p = 0.76) and a statistically significant reduction form baseline in the Immunovac VP4 group (from 4.3 [0.7–8.8] mg/L to 0.3 (0.2–5.8) mg/L (p = 0.004). On day 5 the delta (change from baseline) of CPR was − 3 (ranging from − 7.1 to − 0.4) mg/L in the Immunovac VP4 group vs. − 0.1 (ranging from − 0.9 to 3.9) mg/L in the control group (p = 0.01) (Fig. 4).

**Figure 5:**
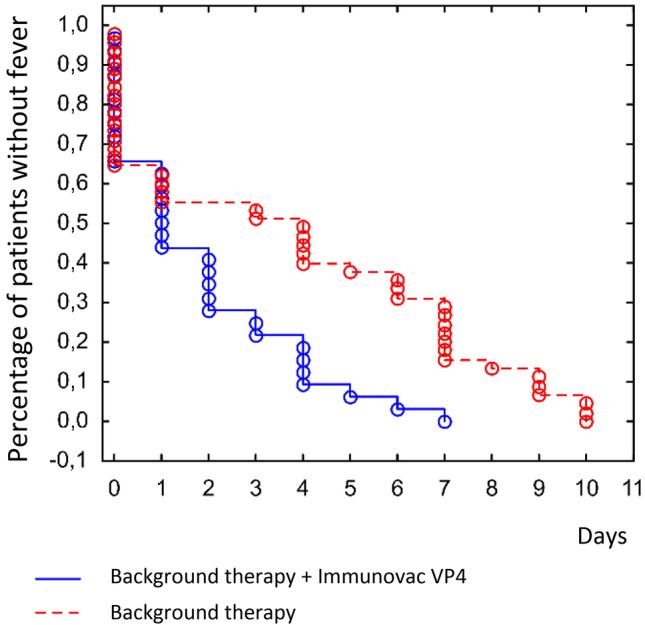
Kaplan–Meier curves for the duration of fever in study subjects with COVID-19.

Analysis of the clinical efficacy of Immunovac VP4 as part of a combination treatment for patients with moderate COVID-19-associated lung disease revealed certain differences. The median duration of fever was estimated using the Kaplan–Meier test. In the Immunovac VP4 group fever persisted for a shorter period than in the control group: 1 (0.5–2) day vs. 4 (1–7) days (p = 0.002) (Fig. 5).

The duration of hospital stay was also shorter in the Immunovac VP4 group than in the control group: 16 (13–19) days vs.19 (16–22) days, p = 0.03.

Assessment of Immunovac VP4 tolerability showed that after subcutaneous administration a red area (hyperemia) was observed at the injection site in 16 patients; it measured 2.5–5.0 cm in diameter in 10 subjects (31.2%) and 5.1–10.0 cm in diameter in 6 patients (18.8%) and did not persist for more than 3 days. There were no reported local infiltrates caused by injections. Systemic reactions in the Immunovac VP4 group included only fever of 38–38.4 °C in three patients (9.3%) and 38.5–38.9 °C in one patient (3.1%), which was similar to the rates in the control group (9.3% and 3.1%, respectively). Fever did not persist for longer than one day and did not require any medication treatment as well as in case of local reactions. Intranasal administration of Immunovac VP4 was not associated with any local mucosal reactions in the upper airways.

## Discussion

The 2009–2010 pandemic caused by the pandemic influenza strain gave a new impetus to the development of new adjuvant vaccines. The next pandemic caused by the spread of SARS-CoV-2 reignited research efforts to develop mucosal vaccines. Studies showed that intranasal vaccines induce high levels of neutralizing antibodies, promotes systemic and local IgA and T-cell responses and almost entirely prevents SARS-CoV-2 infection in both the upper and lower respiratory tracts^28–33^. Immunization at mucosal sites is believed to provide a better virus clearance from the airways and thus prevent its transmission to surroundings^34^. Therefore, in the future immunobiological medications that activate mucosal immunity can be viewed as a promising tool for the prevention of respiratory infections.

A new research direction could involve developing immune-active agents to be used in the phase of active inflammation, when SARS-CoV-2 begins spreading systemically. For example, vaccines containing bacterial ligands have long been used to restore mucosal immune function and thus to prevent complications of respiratory infections^35–40^. Consequently, acting as natural immunomodulatory agents, bacterial vaccines not only induce impaired innate and adaptive immunity, but also suppress excessive immune reactions.

In the conducted study in the group of patients with the addition to basic therapy of the immunotropic bacterial based vaccine in combined regimen of IN and SC administration, it was shown that salivary sIgA levels remained high throughout the active phase when the patients received treatment and within two weeks following discharge (on day 30 of the study) and did not differ from the values seen on admission. It means that despite the cessation of SARS-CoV-2 shedding, improvement in the course of the disease, and amelioration in clinical signs, inflammation was not fully resolved. Saliva is a composite biomarker which not only reflects the state of local immunity (as sIgA levels which we focus on in our studies), but also helps to assess a systemic immune response^41^.

Since we have already previously conducted a study of the assessment of mucosal immunity in IN—per os use of the Immunovac VP4 vaccine^19^, we were interested which way of administration of the drug is the most effective in the complex treatment of patients with COVID-19 associated lung lesions of moderate severity. The obtained results indicate that the levels of sIgA in the secretions of the salivary glands when prescribed an immunotropic drug according to IN and per os regimen during the entire observation period remain in high values: at the beginning of hospitalization 168.7 (95.8–233.8) mcg/l and 140.6 (86.4–213.4) and 154.6 (121.0–200.3) mcg/l after 14 and 30 days from the start of the vaccine, respectively. sIgA levels did not differ from those in group of patients without prescription of Immunovac VP4: 130.6 (93.2–181.2) mcg/l and 126.7 (99.9–164.3) mcg/l on the 14th and 30th days. During the whole period of observation this indicator was statistically significantly higher than the median reference value (71.7 mcg/l) in healthy. So the differences in the dynamics of sIgA level in secretions of the salivary glands under the combined IN and per os administration of the immunotropic drug were not revealed.

Pharyngeal sIgA levels in COVID-19 patients were similar to those in healthy subjects with no history of SARS-CoV-2 infection or exposure to SARS-CoV-2 both in the active phase of the disease and after discharge from hospital. So, we did not observe any changes in sIgA levels in the inflammatory phase, however, the levels of these immunoglobulins changed in patients who were immunized with the vaccine containing bacterial ligands. In this study group, sIgA levels measured on day 30 of the study were higher than in patients receiving only background therapy and even those in healthy subjects; they were also higher than at baseline, i.e. on admission to hospital.

It is important to note that similar changes in the increase of sIgA in the pharyngeal scraping were established in the group of patients with COVID-19 who received the bacteria-based vaccine IN-per os and its values differed from the group of patients without the use of a bacterial ligand. So on the 30th day from the start of its use, this indicator was 29.8 (3.6–106.8) μg/l and 2.9 (0.4–14.8) μg/l, respectively, p = 0.05. Therefore, the use of the Immunovac VP4 vaccine both IN-SC and IN-per os as a part of the complex treatment for COVID-19 patients promotes the production of sIgA in the pharyngeal compartment, which will later highly likely decrease the susceptibility to other respiratory pathogens.

An evident trend was observed in nasal sIgA levels which were high at baseline and by day 30 gradually returned to normal, i.e. the levels seen in healthy subjects with no history of exposure to SARS-CoV-2 (unvaccinated, not infected). In contrast, in the group of patients receiving Immunovac VP4 in combination with basic therapy, on the contrary, an increase in sIgA content was detected by day 30 compared to the baseline, up to 107.0 (44.9–164.5) μg/l, exceeding the values of 30.2 (7.6–61.7) μg/l, p = 0.002 in the group of patients who had only basic therapy. By the way, such dynamics can be seen in patients with the same severity of the disease, but with a combined IN-per os regimen of prescribing an immunotropic drug, where the sIgA count in nasal scrapings increased on day 30 (113.4 (39.8–156.7) μg/l and is higher than in patients without inclusion of bacterial ligands in complex treatment − 37.3 (8.4–66.9) μg/l, p = 0.05. This indicates that regardless of method of prescribing Immunovac VP4 in patients with COVID-19 activation of sIgA secretion in nasal secretions is observed.

Consequently, in patients with moderate severity of COVID-19, the recovery process was accompanied by an increase in the level of sIgA in the nasal and pharyngeal locus on the 30th day of observation, which may subsequently affect both the recovery process and a decrease in the frequency of recurrent diseases. The work of Kryukova et al. showed that the administration of Immunovac VP4 to medical workers who had previously suffered COVID-19 (1st group) was accompanied by an increase in the sIgA level in scraping samples from the oropharyngeal mucosa on the 20th and 90th day up to 19.6 [6.0; 83.8] µg/l and 13.6 [7.4; 48.3] µg/l, respectively, compared to medical staff from the 2nd group without prophylactic administration of the immunotropic drug (4.9 [1.9; 7.6] μg/l (p < 0.05) and 1.8 [1.3; 24.7] μg/l (p < 0.05)^42^. It should be noted that the mechanism of action of the Immunovac VP4 vaccine is based on the activation of innate immune effectors and programming the differentiation of T-lymphocytes to Th1 type. In previous studies of this drug, the following effects were noted: normalization of the number and functional activity of lymphocyte subpopulations (CD3, CD4, CD8, CD16, CD72); programming of proliferation and activation of CD4 T lymphocytes on Th1 pathway; correction of the synthesis of immunoglobulin isotypes towards a decrease in IgE and an increase in IgG, IgA, sIgA^43^.

It is known that secretory IgA is the main class of antibodies present on mucosal surfaces and produced by local plasma cells mainly in the form of dimeric IgA. This subclass of immunoglobulin plays an important role in early protection against respiratory pathogens and is one of the main components of mucosal immunity. Isho B. et al. identified an increase in sIgA to the receptor-binding domain of S protein in saliva samples from patients with COVID-19 after an illness during a long period of time (up to 115 days) compared to controls^44^. Maintaining the level of total sIgA and its increase during Immunovac VP4 administration may indicate the immunoregulatory effect of the drug on the humoral component of mucosal immunity, including a possible increase in the level of specific neutralizing antibodies after an infection.

In the study of Fang L. et al. it was found that daily use of bacterial lysate OM-85 reduced the expression of angiotensin-converting enzyme type 2 receptor and other cell membrane proteins that play a role in SARS-CoV-2 attachment and infection of human epithelial cells. A decrease in the expression of heparan sulfate, which is also a component necessary for the virus to infect cells, was also shown. The described effects of OM-85 on membrane proteins of epithelial cells and specific glycosaminoglycans may explain the reduction in infection of epithelial cells by the SARS-CoV-2 S protein^45^. Probably such changes in immunocompetent cells can also be observed with the use of Immunovac VP4, which requires further research.

The assessment of tolerability of IN-SC administration of Immunovac VP4 used as part of a combination treatment for patients with moderate COVID-19-associated lung disease showed that its administration from day 1 to day 11 of hospital stay was associated only with local injection-site reactions (skin hyperemia that resolved without any medication treatment) during 1–3 days after in 50% of the patients. It was impossible to assess the rate of systemic reactions because the rates of fever were similar (12.4%) in the patients who received and did not receive Immunovac VP4 as part of a combination treatment regimen. The study of safety of Immunovac VP4 confirms previously obtained data that none of the adverse events required discontinuation of the drug course^42^.

Evaluation of clinical markers of effectiveness of Immunovac VP4 for IN-SC administration showed that in the Immunovac VP4 group the patients experienced a more significant reduction in inflammation on day 5, as seen by a statistically significant decrease in CRP, reduction in the duration of fever and the length of hospital stay. Thus, an analysis of the temperature reaction revealed that with IN-SC regimen of immunotropic drug administration, the duration of fever was also lower (1 [0.5–2] days) than in the control group of patients (4 [1–7] days, p = 0.002 but did not differ significantly from the group of patients receiving another—IN-per os (1 [from 0.5 to 2] day)—method of admission^19^. Comparing the duration of hospitalization stay (16 [13 to 19] days) administrating Immunovac VP4 in a combined IN-SC regimen with IN-per os use (16 [11 to 20] days) no differences were identified. However, in both groups of patients who used the immunotronic drug the duration of hospitalization was lower than in the control group (19 [16 to 22] days (p = 0.03)).

## Conclusion

Prescribing of thean immunostimulant agent containing bacterial ligands (Immunovac VP4) in the IN-SC regimen as part of a combination treatment for COVID-19 patients is associated with a gradual increase in the production of sIgA in the nasal and pharyngeal compartments compared to its baseline intensity, which accounts for peaked levels of these immunoglobulins at week 2 after discharge from hospital (30 days from starting the drug). It also reduces the CRP level and shortens the duration of fever and the time to recovery. The results obtained from studying sIgA from the mucous membranes of the upper respiratory tract of patients with moderate COVID-19 are comparable to those obtained when the bacterial lysate was administered intranasally and per os. Therefore, both regimens of administration of an immunotropic drug can be used in this cohort of patients. It is possible that the activation of the synthesis of the secretory component IgA in these loci of the respiratory tract accompanied by activation of not only immune mechanisms of mucosal, but also systemic resistance that should be further investigated.

## Data Availability

The datasets generated during and/or analysed during the current study are available from the corresponding author on reasonable request.
